# Development of a Function-Integrative Sleeve for Medical Applications

**DOI:** 10.3390/s19112588

**Published:** 2019-06-06

**Authors:** Moritz Neubauer, Eric Häntzsche, Christina Pamporaki, Graeme Eisenhofer, Martin Dannemann, Andreas Nocke, Niels Modler, Angelos Filippatos

**Affiliations:** 1 Institute of Lightweight Engineering and Polymer Technology (ILK), Technische Universität Dresden, 01307 Dresden, Germany; moritz.neubauer@tu-dresden.de (M.N.); martin.dannemann@tu-dresden.de (M.D.); niels.modler@tu-dresden.de (N.M.); 2 Institute of Textile Machinery and High Performance Material Technology (ITM), Technische Universität Dresden, 01307 Dresden, Germany; eric.haentzsche@tu-dresden.de (E.H.); andreas.nocke@tu-dresden.de (A.N.); 3 Department of Medicine III & Institute of Clinical Chemistry and Laboratory Medicine, Medical Faculty and University Hospital Carl Gustav Carus, Technische Universität Dresden, University Hospital Carl Gustav Carus at the Technische Universität Dresden, 01307 Dresden, Germany; christina.pamporaki@uniklinikum-dresden.de (C.P.); graeme.eisenhofer@uniklinikum-dresden.de (G.E.)

**Keywords:** smart textiles, warming device, blood flow, multi-material design, function integration, phaeochromocytomas/paragangliomas (PPGLs), medical engineering

## Abstract

Function-integrative textiles bear the potential for a variety of applications in the medical field. Recent clinical investigations suggest that the application of a function-integrative fabric could have a positive impact on currently applied diagnostic procedures of a specific type of tumour. In this context, the fabric should enable local warming of a patient’s upper extremity as well as blood flow measurement. Existing solutions comprise a warming system but lack a measuring apparatus for blood flow determination. With regard to the quality of results of current diagnostic procedures, the local warming of the patients’ upper extremity and the simultaneous determination of the blood flow plateau are crucial. In the present paper, the development process of a function-integrative sleeve is introduced. Besides the development of an adaptable sleeve-design, the manufacturing process of an integrated warming system was also addressed. Furthermore, the identification of crucial physiological effects, using a Laser Doppler Perfusion Monitor, is introduced. During testing of the function-integrative sleeve, modulation of the desired physiological effects was observed. The results support the initial assumptions and dictate further investigations on increasing user-friendliness and cost-efficiency during adjusting and determining the physiological effects in the course of tumour diagnosis.

## 1. Introduction

Phaeochromocytomas/paragangliomas (PPGLs) are catecholamine-producing tumours arising from chromaffin cells of the adrenal medulla or extra-adrenal paraganglionic tissue. They account for up to 0.6% of cases of adult and 1% of paediatric hypertension [1]. Although a rare cause of secondary hypertension, with a reported annual incidence of 2–5 cases per million, of which only 10% occur in children [2,3,4], PPGLs are potentially lethal. Therefore, confirmation or exclusion of these tumours among patients with secondary hypertension is of paramount importance. As clinical presentation of PPGLs can be varied with nonspecific signs and symptoms, the diagnosis is crucially dependent on measurements of plasma free normetanephrine and metanephrine, the O-methylated metabolites of noradrenaline and adrenaline [5,6,7,8]. Various factors before and during blood sampling such as posture and variations in ambient temperature [9,10] have been shown to impact the diagnostic performance of plasma free metanephrines, resulting in higher rate of false positive results. In such cases, patients have to deal with time consuming and costly follow-up examinations, not to mention psychological stress. Based on the above considerations, it was recently shown that local warming of the forearm results in reduced plasma free normetanephrine levels and therefore lower rates of false positive results [10,11].

This paper describes the development process of a function-integrative sleeve. The term function-integrative combines the possibility to include the functions of adjusting the blood flow of a patient’s upper extremity to maximum vasodilation, enabling blood flow measurement with the help of a Laser Doppler Perfusion Monitor as well as to access the vein for blood sample withdrawal. A device with these mentioned functions could be of high value concerning the number of false-positive results of the biochemical diagnostic procedure for the determination of PPGLs and consequently the reduction of personnel costs. By enabling “arterialisation” of venous blood through local warming, the technical device can also be useful in other fields of medical care such as in intensive care unit practices, where for instance in various cases injection of medication is preferred in arterial than venal vessels. In addition to the design and manufacturing of a prototype, an approach for the offline-identification of the point in time when the maximum vasodilation is reached was developed and verified by means of a function test of the prototype.

To develop a function-integrative sleeve for improving existing diagnosis procedures of PPGLs, a basic understanding of the underlying physiological processes and existing technical solutions is necessary.

### 1.1. Noradrenaline Kinetics

Plasma concentrations of noradrenaline in forearm venous outflow are derived from two sources: noradrenaline from the arterial inflow that escapes local extraction (mainly by extraneuronal monoamine transporters) and transmitter released from sympathetic nerves of the forearm that escapes local uptake processes (mainly by neuronal transporters) to enter the venous drainage [12,13,14,15]. Concentrations of both are influenced by changes in local blood flow. In particular, reduced blood flow due to vasoconstriction leads to increased extraction of noradrenaline from the arterial inflow before it enters the venous outflow. Under these circumstances, the result is an increased proportion of noradrenaline in forearm venous blood from locally released compared to systemic sources. Vasoconstriction-associated increased local release of noradrenaline from forearm sympathetic nerves may further contribute to an increased proportion of locally-released to systemically derived noradrenaline in forearm venous plasma. Furthermore, the reduced venous blood flow into which escaping noradrenaline is diluted can also contribute to increased forearm venous plasma concentrations of the amine.

Normetanephrine and metanephrine, the respective O-methylated metabolites of noradrenaline and adrenaline, are cleared rapidly from the circulation by the same extraneuronal monoamine transporters responsible for the circulatory clearance of catecholamines [16,17]. Normetanephrine in forearm venous plasma, similar to noradrenaline, has two sources including that entering from the arterial inflow and which escapes local extraction and from noradrenaline locally released in the forearm [16]. Thus, forearm venous normetanephrine is influenced by changes in blood flow in the same way as noradrenaline. In contrast, the local forearm kinetics of adrenaline and metanephrine are much simpler; differences in concentrations of these amines in venous outflow compared to the arterial inflow almost completely reflect local extraction.

As we have shown previously, and in line with the above considerations, normetanephrine but not metanephrine shows increases in forearm venous plasma concentrations in colder winter than warmer summer months as well as decreases during warming of the forearm [10]. Consequently rates of false-positives for plasma normetanephrine are higher in winter than summer months. Interestingly, during warming of the forearm in patients with PPGLs, plasma concentrations of normetanephrine increased rather than decreased. This likely reflects decreased forearm extraction of normetanephrine from the arterial inflow and in patients with PPGLs the predominant source of the amine from tumoral release into the systemic circulation rather than from noradrenaline after release and metabolism to normetanephrine within forearm tissues.

The above results imply that warming of the forearm in patients tested for PPGLs should result in an increased signal strength for forearm venous plasma concentrations of normetanephrine in patients with compared without tumours, a result of both increased concentrations in patients with tumours and decreased concentrations in patients without tumours. Taking into account that normetanephrine is the most important metabolite for the diagnosis of PPGLs, blood sampling under conditions of forearm warming should be expected to improve the diagnostic performance of the test and in particular minimise false-positive test results.

### 1.2. Physiology of Warming Induced Vasodilation of the Forearm

**Figure 1:**
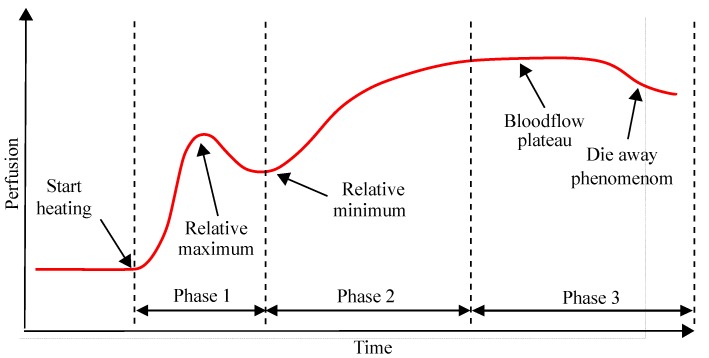
Skin perfusion as a function of time during warming of the considered body portion according to Kellogg et al. [19].

In healthy volunteers, local warming of the upper extremity leads to an increase in local blood flow to a maximum, defined as the blood flow plateau. For patients who are not suffering from vasomotor dysfunction, the blood flow plateau occurs after 30–45 min of continuous warming of the upper extremity [18,19]. The characteristic course of the blood flow over time during uniform warming of the upper extremity is shown schematically in Figure 1. When a warming stimulus is applied on the forearm, the flow velocity of the peripheral blood initially increases to a relative maximum for 2–3 min and then decreases until a relative minimum is reached (Phase 1 in Figure 1). After the relative minimum, the blood flow velocity increases again until it reaches a nearly constant flow velocity (Phase 2 in Figure 1), the so-called blood flow plateau [18,19]. The blood flow plateau characterises the stage of maximum vasodilation and reduced activity of sympathetic nervous system (Phase 3 in Figure 1) [11,12,14]. Furthermore, it has been observed that after prolonged warming of the upper extremity, a phenomenon known as “die away”, occurs (Phase 3 in Figure 1). This phenomenon, in which blood flow slowly returns to its starting point, usually occurs about 45 min after the onset of warming. In this context, it is important to note that in patients with significant vasomotor dysfunction in diabetes or other disease states [20,21], often a lower highest possible vasodilation level is observed, possibly occurring before the expected time of 30–45 min [22,23]. Consequently, in this group of patients, the premature occurrence of a “die away” phenomenon could affect the informative value of the biochemical analysis of a blood sample since the blood flow plateau and its associated favourable physiological conditions would be missed if blood sampling was performed at between 30 and 45 min.

Given these above physiological facts, the use of a conventional warming pad is insufficient, as it is not possible to determine whether and when the maximum vasodilation occurs for each specific patient. Consequently, simultaneous warming of the upper extremity and blood flow measurement for each individualised patient are crucial to determine the most optimal conditions and time for blood sampling and improve the diagnostic performance of the test [11].

### 1.3. Current Approaches for Stimulating and Measuring the Blood Flow

According to the current state of the art, there are various approaches for adjusting the maximum blood flow of a human by applying a warming stimulus. Existing applications with integrated fluid and pressure chambers allow the increase of peripheral blood flow due to the heat dissipation of the fluid [24,25,26]. Furthermore, there is a broad spectrum for applications for enhancement of blood circulation comprising resistive heating elements [27,28]. In addition, various functional textile applications for medical use were introduced by Stoppa and Chiolerio [29]. However, according to the state of the art, there is no device comprising the function of forearm warming, blood flow measurement and determination of the blood flow plateau.

To monitor the rise in blood flow caused by forearm warming, an in vivo measurement method can be applied. The most commonly employed techniques for the in vivo measurement of arterial blood flow in individual organs involve the use of flow probes or sensors. Commercially available systems for the measurement of in vivo blood flow can be divided into two categories: ultrasonic and electromagnetic [30]. The application of a laser-based measuring device in recent studies, investigating the correlation between the rise in forearm surface temperature and blood flow, suggest its application for the present investigation. In this study, a Laser Doppler Perfusion Monitor (LDPM) of the type PERIFLUX SYSTEM 5000 of the company PERIMED was used [31]. Laser Doppler Fluxometry uses the laws of the laser-Doppler principle to determine the velocities of fluids by frequency change of optical waves. The directionless measurand is called “flux” and is expressed in perfusion units (PU) [32].

## 2. Proof of Concept

Within the scope of the present work, the suitability of carbon fibre-based heating elements with respect to the increase of peripheral blood flow is examined. Compared to most metals, carbon fibres have a high electrical resistance and a high thermal conductivity in fibre direction and are therefore suitable for use as a resistance heating element [33]. The resulting electrical resistance of a carbon fibre heating element $R_{HE}$ depends on the specific electrical resistance ρ of the material as well as the length *l* and the cross-sectional area, which depends on the diameter $d_{Fil}$ of the individual filament of the heating elements and their number *k*, as shown in Equation (1),

(1) $$R_{HE}=\rho *\frac{l}{\frac{\pi }{4}*d_{Fil}^{2}*k}.$$

Carbon fibre heating elements (HE) can generate heat uniformly and rapidly and have a high corrosion resistance which predestines it for realising heating structures. The combination of the high strength and rigidity with the small diameter of only 7 μm leads to a flexible material behaviour of carbon fibre (CF) suitable for clothing applications [34,35]. Current applications of heating garments comprise a variety of methods for electrical contacting of the HE including crimping, conductive adhesives, embroidering as well as a combination of these methods [36,37]. Throughout the manufacturing process of the functional warming layer, a variation of materials and methods were tested in order to achieve a fast and reproducible manufacturing process and a non-obtrusive application suitable for clothing.

Studies observing the relationship between skin temperature and blood flow show that a maximum increase in forearm blood flow occurs, when the surface temperature of the upper extremity is increased to 42 ${}^{\circ }$C and maintained at this temperature level [38,39,40]. The required heat output ${\dot{Q}}_{HE}$, in order to increase the skin surface temperature to 42 ${}^{\circ }$C, was calculated by Equation (2). The considered heat flows are the dissipated heat output due to thermal radiation and heat transfer ${\dot{Q}}_{HT}$ as well as the thermal input of the human body ${\dot{Q}}_{Arm}$. The heat radiation ${\dot{Q}}_{TR}$, where $A_{W}$ is the surface area, $\phi_{1,2}$ represents the view factor from surface 1 to surface 2, σ is the Stefan–Boltzmann constant and $\theta_{1}$ is temperature of the sleeve surface and $\theta_{2}$ the surface temperature of an object close to the patient, is calculated by Equation (3). The heat transfer is determined based on the calculation equation for the total heat flow of a multi-layer pipe. It should be noted here that the complex thermodynamic processes within the blood circulation are not taken into account. Consequently, the heat transfer from the blood circulation to the skin layer is not considered and the skin layer on the inside is considered to be perfectly insulated. The calculation of the heat loss on the patient’s arm is carried out taking into account the skin temperature $\theta_{S}$, the ambient temperature $\theta_{A}$, the arm length $L_{Arm}$ and the heat transfer coefficient *k* by Equation (4). The heat output, which is generated by metabolic processes in the body and released via the arm surface $A_{Arm}$ of the patient, is calculated from Equation (5). The assumption is made that there is an even heat dissipation $Q_{B}$ related to the body surface area $A_{B}$.

(2) $${\dot{Q}}_{HE}={\dot{Q}}_{HT}+{\dot{Q}}_{TR}-{\dot{Q}}_{Arm}$$

(3) $${\dot{Q}}_{TR}=\phi_{1,2}\cdot \sigma \cdot A_{W}\cdot \left(\theta_{1}^{4}-\theta_{2}^{4}\right)$$

(4) $${\dot{Q}}_{HT}=k\cdot L_{Arm}\cdot \left(\theta_{S}-\theta_{A}\right)$$

(5) $${\dot{Q}}_{Arm}={\dot{Q}}_{B}\cdot \frac{A_{Arm}}{A_{B}}$$

The necessary heat output of ${\dot{Q}}_{HE}=26.8\;W$ was analytically calculated based on Equation (1) by the sum of ${\dot{Q}}_{HT}=37.3\;W$ and ${\dot{Q}}_{TR}=4.5\;W$ minus the ${\dot{Q}}_{Arm}=15\;W$ generated by the body. The calculated value of ${\dot{Q}}_{HE}$ represents a guide value for the design of the functional heating layer. However, an increase of the heating power to 35 W seems reasonable, since the cooling influence of the blood circulation was not taken into account in the analytical calculation. The danger of overheating is excluded by the provided device for temperature control. Thus, an increased heating power only results in a reduced heating time. At this point, it should be pointed out that the calculated heat output should enable warming of a patients arm in order to achieve the favourable physiological conditions for each case and not necessarily within the same duration. Therefore, an adjustment of the imported heat output was not considered necessary based on the mass of the arm.

**Figure 2:**
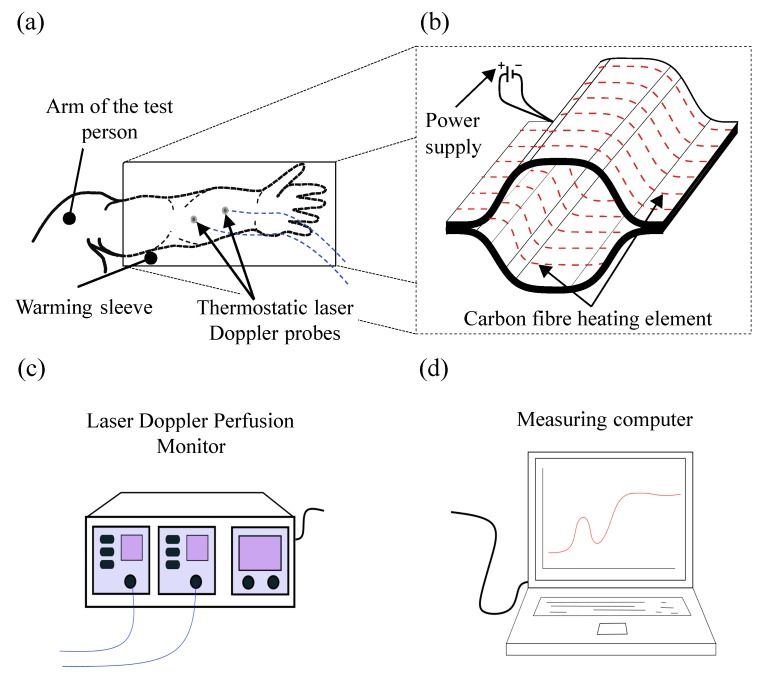
(**a**) Positioning of the warming sleeve and the measuring probes on the patient’s arm; (**b**) detailed view of the warming sleeve; (**c**) data processing; and (**d**) evaluation of the blood flow curve.

To determine whether CF-based HE produce the desired physiological effects on the human body, a proof of concept was carried out using a warming sleeve (Figure 2b). In this context, an increase of the local blood flow at the patient’s arm to the level of the blood flow plateau is the decisive criterion that has to be fulfilled. Consequently, to increase the surface temperature of the human skin to 42 ${}^{\circ }$C and achieve a higher blood flow, the warming system Yorbay 1 by the company Yorbay was selected, delivering a thermal output of 35 W at an operating voltage of 12 V. The HE of this System consists of 3000 (3k) single filaments and is mainly used for warming seats in passenger cars. The corresponding electrical resistance results from Equation (1). For the evaluation of the concept, two of the warming systems are connected so that they form an arm-length sleeve shown in Figure 2b. The patient’s arm is inserted into the warming sleeve. Subsequently, the skin surface is warmed to 42 ${}^{\circ }$C. The blood flow and the temperature of the skin surface is measured by the Thermostatic Laser Doppler Probes as shown in Figure 2a. The evaluation of the measurement results is carried out using the software-tool PERISOFT provided by PERIMED and is shown in Figure 2d [31].

**Figure 3:**
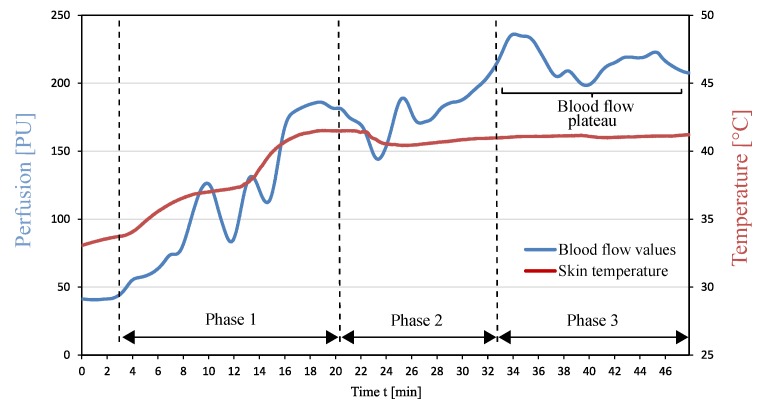
Blood flow during local warming of the forearm.

In Figure 3, the values of the blood flow measurement and the temperature of the skin surface of a test person are plotted over the measurement period t. The blood flow values in Figure 3 show a clear increase over the measured time period. At the beginning of local warming, the blood flow value is 47 PU. In Phases 1 and 2, more than one relative minimum and maximum were observed, which is due to movement of the forearm and physiological effects. However, as shown in the last section of the diagram, after a warming period of 34 min, the blood flow reached a maximum of 235 PU. In the further course of the measurement, the blood flow averages 210 PU at an approximately constant temperature of the skin surface of 42 ${}^{\circ }$C. On the basis of previous blood flow tests, the blood flow values recorded in the time range t = 33 min until the end of the measurement are assigned to the blood flow plateau. The occurrence of this anticipated physiological effect proves the function of the investigated warming concept. At this point, it should be pointed out that the definition of the blood flow plateau does not depend on the occurrence of the die away phenomenon. It can be primarily recognised as a relative steady perfusion after the observation of a relative maximum. The goal of achieving the blood flow plateau is based on the fact that in that time period we have high perfusion for a relative long duration. Since we observed a fourfold increase of the perfusion and achieved these high values for a period of 13 min and based on medical experience, the observed high blood values correspond to the blood flow plateau. This is due to the underlying physiological mechanism, where the observation of the relative maximum is followed from a small increase and then from a steady blood flow.

## 3. Development of the Function-Integrative Sleeve

To realise the approved concept, a prototype of a function-integrative sleeve was developed. For this purpose, a functional design of the sleeve as well as the design of a warming system and the geometric arrangement of the comprised HE were determined.

### 3.1. Design of the Function-Integrative Sleeve

In the first step, the design of the sleeve was determined and the functional details and dimensions were defined aiming on user-friendliness and functionality. The criteria shown in Table 1 were defined on the basis of functionality requirements regarding the sleeve described in [11]. Furthermore, the geometric dimensions were defined on the basis of an anthropometric database, which provides the considered arm dimensions of woman and men as well as the corresponding percentiles [41,42]. In this context, the objective was to define the geometric dimensions in such a way that as many people with different arm dimensions as possible could potentially wear the sleeve.

**Figure 4:**
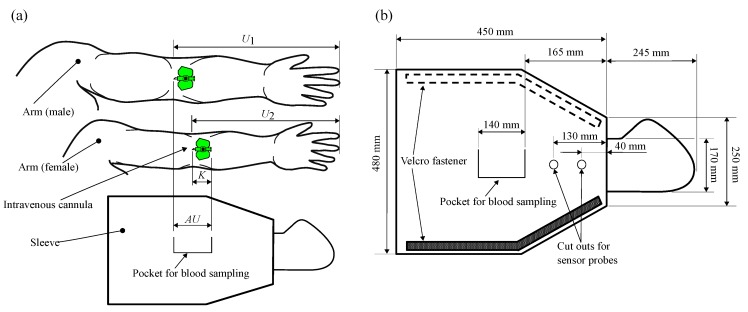
(**a**) Method of dimension definition for the function-integrative sleeve; and (**b**) dimensions and functional details.

Criterion 1 requires that the prototype of the function-integrative sleeve must cover the entire arm up to the shoulder of the patient. To allow a facile adaptation of the sleeve to the patient’s arm circumference, a sleeve-shaped design was chosen (Criterion 2). To minimise a restriction of finger motion, the sleeve was designed as a mitten in the hand area (Criterion 3). The cut outs for the sensor measuring heads of the LPDM are provided below the mitten at the level of the forearm (Criterion 4). In the area of the arm bend, a pocket with a closure system is provided for retrieving blood samples (Criterion 5). The process of determining all required dimensions is described by defining the length AU of the pocket required for blood collection based on a literature research on anthropometry [41,42] (Criterion 6). In the further course of designing the sleeve, the measurements referring to the 95th percentile of men were defined as the maximum reference values and the 5th percentile of women to the minimum reference values. The definition of the dimensions and measurements based on these reference values allows a wear-ability for up to 90% of the population. Figure 4a shows the arm of a man whose forearm length $U_{1}$ refers to the 95th percentile and thus defines the statistical maximum value. The arm shown below represents the arm of a woman whose forearm length $U_{2}$ lies in the range between the 5th and 50th percentile defining the minimum forearm length. To enable blood sample collection from all test persons whose forearm length lie in the range of $U_{1}$ (upper limit value) and $U_{2}$ (lower limit value), the length of the pocket was calculated according to Equation (6),

(6) $$AU=(U_{1}-U_{2})+K.$$

In the further course of defining the dimensions of the sleeve, the explained method for determining the measurements was retained. The important dimensions of the sleeve as well as the functional solutions, which were defined according to the criteria in Table 1, are presented in Figure 4b.

### 3.2. Design and Arrangement of the Heating Elements

**Figure 5:**
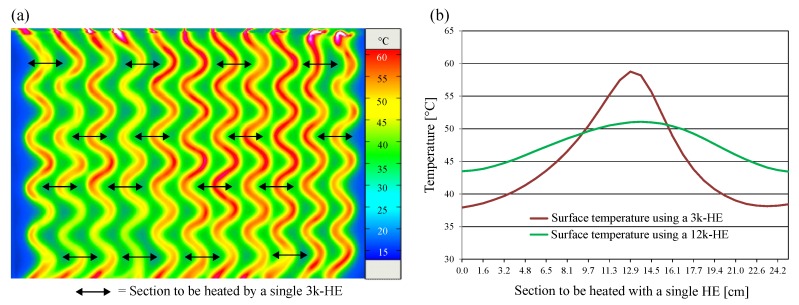
(**a**) Thermogram of the textile surface of the sample warming system with 3k-HE; and (**b**) comparison of the experimentally determined surface temperatures using 3k- and 12k-HE.

The 3k-HE used for the proof of concept in Section 2 generated an inhomogeneous temperature distribution throughout the surface of the warming sleeve. Therefore, the objective was to minimise differences in temperature distribution across the effective width to be heated by one HE. With a constant heat output, an increase in the number of filaments per HE causes a reduction in the heat output per filament. Consequently, the temperature of the individual filament was reduced. To determine the influence of the number of filaments on the heat distribution throughout the warming layer, a comparison between a 3k-HE and HE with a higher number of filaments was performed. To reduce the scope of experimental investigations, a prior numerical investigation was performed. The numerical investigation implied that temperature distribution homogeneity could be significantly increased using a 12k-HE. Consequently, a sample warming system with a 3k-HE and another one with a 12k-HE were manufactured and tested. The HE of the manufactured sample warming system shown in Figure 5 are shorter in length compared to those of the final application, which results in higher absolute surface temperature values than the maximum permitted temperature of 42 ${}^{\circ }$C. At a constant heat output of 30 W, the heat distribution of both systems was measured over 16 sections to be heated by a single HE, as shown in Figure 5a. The averaged temperature curves of the experimentally determined data shown in Figure 5b establish that the use of the 12k-HE lowers the maximum value of the surface temperature significantly. Furthermore, an increased homogeneity of the temperature distribution over the section to be heated by a single HE, compared to the 3k-HE, was achieved. Consequently, the 12k-HE was selected for the manufacturing of the functional warming layer of the sleeve.

**Figure 6:**
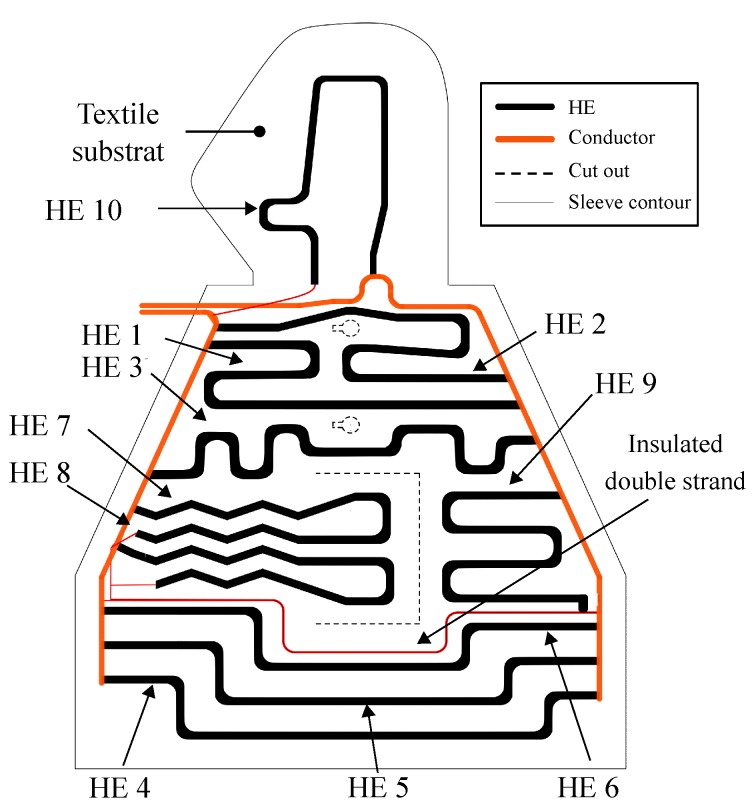
Geometric arrangement of the components of the functional warming layer.

To arrange the 12k-HE, the restrictions of the selected geometry of the sleeve, the cut-outs of Laser Doppler Probes and the pocket for blood sampling, must be taken into account. As the homogeneity of the temperature distribution increases with the number of the heating elements, the respective effort rises regarding the manufacturing process (contacting, positioning and fixation of the HE). In this context, throughout the investigations the number of 10 heating elements was determined as a reasonable compromise between manufacturing effort and homogeneity of temperature distribution. Furthermore, the heating elements had to be contacted with the conductor strands. The strands were positioned on both sides of the sleeve to exclude them from excessive bending caused by the movement of the upper extremity. By the aim of reaching an equal distance between the HE to ensure homogeneous heat distribution and minimising the manufacturing effort, the 12k-HE with a length of 0.5 m were arranged in an iterative process. After repeatedly adjusting the geometric arrangement of the HE, the variant shown in Figure 6 was finally selected.

The HE 1–6 run directly from the left to the right conductor. Due to the geometrical restriction, caused by the cut outs for the blood collection pocket and the Laser Doppler Probes, HE 7–10 must be connected to the opposite conductor using additional wiring. The geometry shown was transferred into a vector file for subsequent textile processing.

## 4. Manufacturing Process of the Prototype

The following section comprises the description of the manufacturing process of the three main layers, which are the contact, functional and isolation layer of the function-integrative sleeve. During the process, the emphasis was laid on determining the parameters required for the production of the functional warming layer.

### 4.1. Manufacturing of the Contact and the Isolation Layer

**Figure 7:**
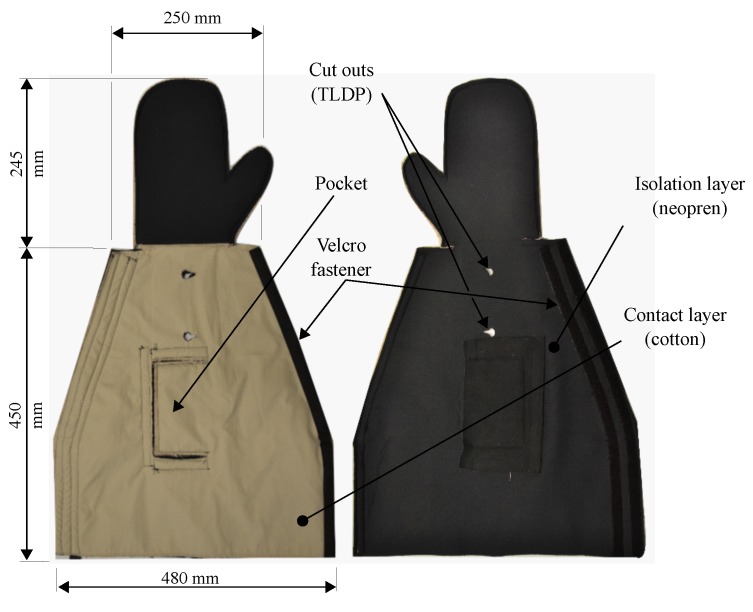
Design prototype of the function-integrative sleeve.

The comprised prototype of a function-integrative sleeve consists of three layers with individual functions and selected materials. The first layer is made out of cotton, shielding the human skin against the direct contact to the functional warming layer. The functional warming layer is made of a heat resistant fleece and works as a substrate for the HE, conductors and sensors. The isolation layer is made of neoprene to ensure thermal isolation for minimising the heat output of the warming layer. To test the dimensions of the sleeve determined in Section 3, the design prototype shown in Figure 7 was manufactured, consisting of the contact and the isolation layer. Subsequent fittings of the sleeve by different persons showed that functionality, such as the collection of blood samples, was not affected by different lengths and circumferences of a test persons arm. Thus, the suitability of the selected dimensions of the sleeve was confirmed. However, the arrangement of the Velcro straps was adjusted to ensure customisability of the sleeve to smaller arm circumferences.

### 4.2. Manufacturing of the Functional Warming Layer

**Figure 8:**
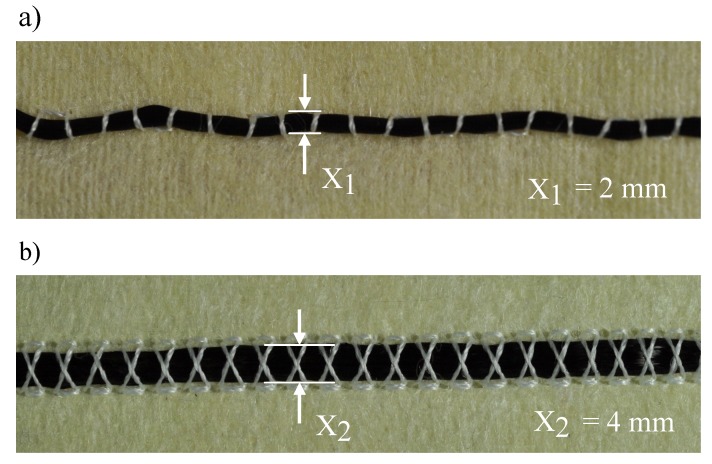
(**a**) Reduced lay-down width of a 12k-HE due to thread-constriction; and (**b**) increased lay-down width of a 12k-HE due to stitch pattern optimisation.

The main focus of the manufacturing process was laid on the attachment of the HE and its corresponding conductor on the chosen heat resistant fleece. For high position reproducibility, the tailored fibre placement method was used for fixation of the components [43]. Thereby, the components can be fixed on a substrate material through the looping of the upper and lower threads. This allows arbitrary geometrical lay-down of the components on the substrate material. The corresponding width of the fixed material on the substrate is defined as the lay-down width. Among other parameters, the lay-down width depends on the thread tension of the upper thread and on the selected stitch pattern. The materials to be fixed, required for the functional warming layer, are the HE, conductors and the insulated twin strand. The first tests showed that the HE is tied together at low yarn tension, resulting in a higher manufacturing speed but a lay-down width of only 2 mm, as shown in Figure 8a instead, of the original width of 7–7.2 mm. With regard to the heat distribution of the HE, a reduction in the lay-down width would result in excessive temperatures, since the heat dissipation of the HE takes place over a smaller area. To prevent this and to ensure that the heat is discharged over a wider area, the cross stitch is applied. Due to the changed stitch pattern, its geometry in combination with an increased thread tension increased the lay-down width of the HE from the original width of 2 mm up to 4 mm, as shown in Figure 8b. The lower manufacturing speed is tolerated in favour of the reduced waviness and maximised lay-down width.

**Figure 9:**
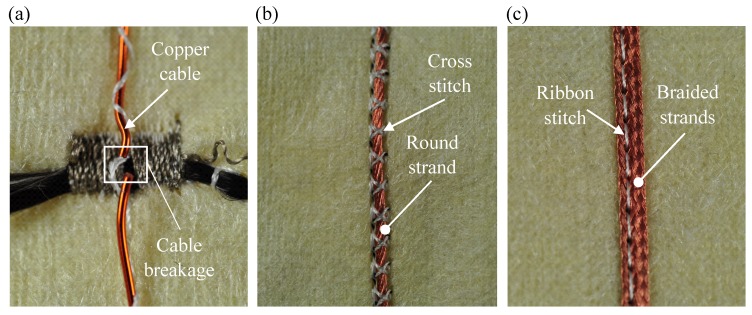
(**a**) Damaged copper cable; (**b**) copper strand fixed by cross stitch; and (**c**) copper braided strand fixed by ribbon stitch.

In the next step, the suitability of the conductor constellation and its placement on the functional warming layer was investigated. Thereby, the main evaluation criteria are flexibility, process capability and the electrical contact resistance $R_{Cont}$ between the HE and the conductor. During pre-investigations, which included the fixing of the material, it was found that the copper cable is unsuitable for the present application. The brittleness and the low flexibility of the material led to a breakage of the cable in the course of the fixation process, as shown in Figure 9a. Due to the resulting restriction the application of copper cables for the functional warming layer is ruled out. In the following tests, the suitability of copper strands was therefore tested. The advantage of a strand conductor is the high flexibility of the individual wires resulting in a shift instead of a breakage caused by the needle. In addition, the flexibility of the entire structure is increased due to the small diameter of the individual wires in the strand compared to the compact copper wire. In comparison to the round strand shown in Figure 9b, the strand braid in Figure 9c can be pierced and fixed in the middle by the ribbon stitch pattern due to the geometric arrangement of the individual wires. Consequently, an increase in positioning accuracy during the embroidery process and a reduction of the number of stitches and thus the stress on the substrate textile could be achieved. To determine the most suitable conductor constellation concerning process- and contact ability, the application of the conventional round strand and the braided strand are compared in the further course of the investigation.

**Figure 10:**
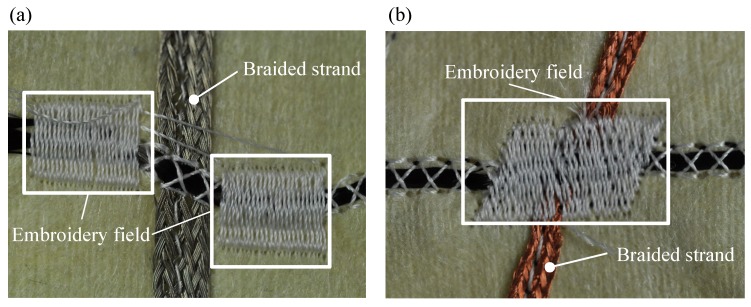
(**a**) Lateral positioning of the embroidery fields; and (**b**) central positioning of the embroidery field above the contact point.

Besides the process of contacting the HE via crimping, where a ferrule is attached on both ends of each heating element using crimping pliers, alternative methods of contacting the HE were tested. To find a way to reduce manufacturing time, increase flexibility of the function-integrative sleeve and improve the adaptability of the sleeve to the upper extremity of the patient. First attempts of contacting the HE with conductive epoxy were not satisfying as the low elasticity of the used adhesive led to a failure of the adhesive bond after several times of adjusting the sleeve to the arm. Consequently, an alternative method was investigated by integrating the contacting process into the manufacturing process of the warming layer. Throughout the first test, embroidery fields were positioned on both sides of the contact point, as shown in Figure 10a, to apply a contact force. Resistance measurements showed that the applied contact force of the embroidery fields is not sufficient to significantly increase the contact area between the conductors. In fact, a low contact resistance ($R_{Cont}$) could not be achieved with the lateral positioning of the embroidery fields. In the next step, the embroidery field is positioned directly on the contact point as shown in Figure 10b. Compared to the lateral embroidery fields, a significant reduction in $R_{Cont}$ was achieved. Consequently, the central positioning of the embroidery fields above the contact point was used for further investigations.

The influence of the strand geometry (round strand or braided strand) was investigated as well as the influence of electrical conductivity (γ) of the upper thread material (conductive or non-conductive) to determine a combination with a minimum $R_{Cont}$. The textile contact combinations were compared to the commonly used contacting process of crimping, which served as a reference. The determined values for $R_{Cont}$ of the investigated combinations, using the four-point probes electrical impedance measuring technique, are shown in Table 2.

**Figure 11:**
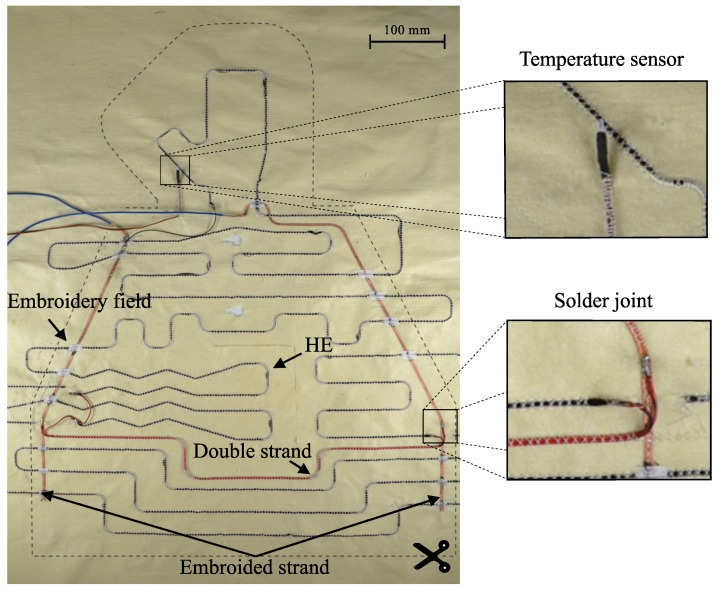
Warming layer with integrated functional components.

The results show that Combinations 3 and 4 comprising textile contacting have a lower $R_{Cont}$ compared to the crimp method. The lowest $R_{Cont}$ was achieved in Combination 3, using electrically conductive upper thread material. However, when the silver-plated thread-material is used, there is an increased tearing of the upper thread in the embroidery process. This effect is attributed to the increased surface roughness of the silver-plated thread. The increasing friction force between the thread and the thread-guide of the machine results in an excessive thread-tension and ultimately leads to the tearing of the thread. For this reason, Combination 4 was selected for the production of the function-integrative warming layer. The slightly higher $R_{Cont}$ is tolerated in favour of the higher process reliability. Subsequently the functional warming layer was manufactured as shown in Figure 11. Due to geometrical restrictions caused by the cut outs for the blood sample pocket and the sensor probes, three HE could not be connected directly to both conductors. Thus, one end of each of these three HE was crimped and contacted with additional cables by soldering.

## 5. Testing of the Prototype

**Figure 12:**
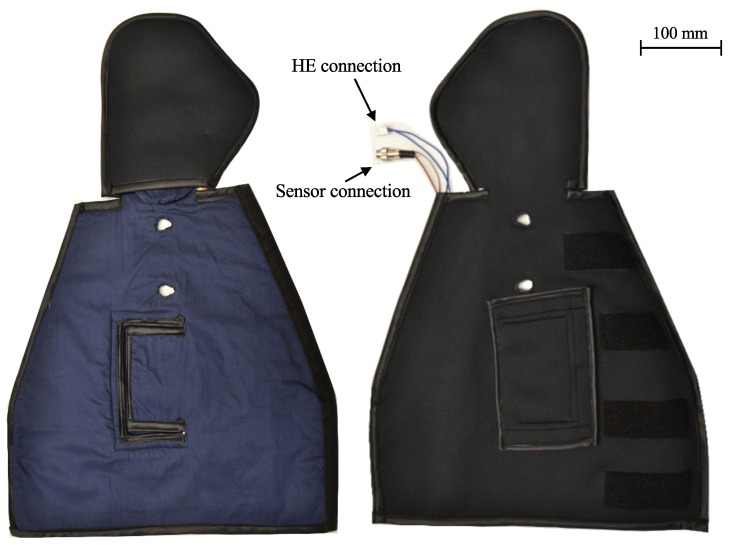
Prototype of the function integrative sleeve.

After the development process of the prototype was completed, the textile assembly of the sleeve took place. The finished prototype is shown in Figure 12. In the following, individual functions of the sleeve were tested. Besides a function test of the sensors and the HE, it was also checked whether the blood flow plateau can be reached using the function-integrative sleeve. Subsequently, an off-line determination of the blood flow plateau, using the approach described in the prior Section 5.2, was performed.

### 5.1. Function Test of the Heating Elements

**Figure 13:**
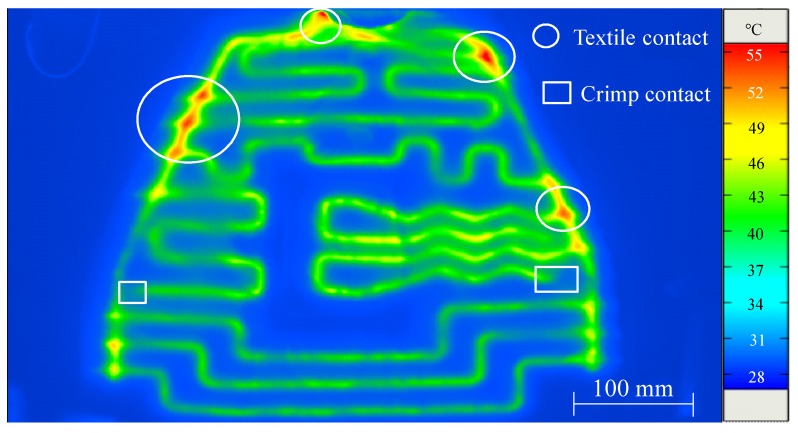
Thermogram of the contact layer surface in the operating state of the functional warming layer.

The functionality of the HE was confirmed as shown from the local increase in temperature on the surface of the contact layer in Figure 13. Figure 13 shows that an excessive increase in temperature is measured at the marked textile contact points of the HE. These “hotspots” are due to an increased electrical resistance of the textile contacting point between HE and braided strand. In contrast, no increased temperature values were measured in the area of crimp contacting. The resistance measurements carried out in Section 4.2, however, yielded lower electrical resistance values for textile contacting compared to crimp contacting. A possible influencing factor on the value of the $R_{Cont}$ could be the degree of surface oxidation of the copper strands, which subsequently contributes to an increase in the impurity layer resistance. Furthermore, it is assumed that a frequent application of the sleeve and the associated stress, results in a reduced thread tension of the textile bond. The reduced contact force leads to a decrease in the conductivity of the contact point and thus to an increase in $R_{Cont}$. With regard to the patient’s safety, uniform temperature distribution is a decisive criterion that must be met by the heating system. Therefore, actions have to be taken to increase the conductivity and the robustness of the contact areas. One possible measure to counteract the increase in $R_{Cont}$ is the use of a silver-containing two-component adhesive in the area of the contact point. In addition, the use of conductive upper thread material could further reduce the $R_{Cont}$. Due to the arising difficulties during manufacturing described in Section 4.2, an adaptation of the manufacturing process would be necessary. The effectiveness of these measures could be investigated within the framework of future optimisation approaches of the sleeve.

### 5.2. Approach for Automated Blood Flow Determination

The motivation behind the development of an approach for an automated detection of the blood flow plateau (BFP) is to minimise personnel costs related to the supervision of the patients’ blood flow and the detection of the blood flow plateau. Furthermore, an accurate automated detection of the BFP could prevent a blood sample collection during the occurrence of the “die away” phenomenon.

**Figure 14:**
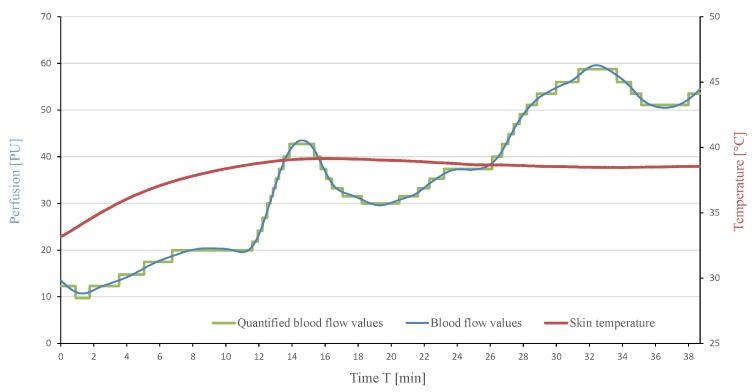
Blood flow values and its corresponding quantified curve.

Since the maximum blood flow values at the BFP vary between patients, the BFP cannot be determined from an absolute value measured. Furthermore, the numerous local maxima and minima of the blood flow curve that occur despite filtering the measurement signal do not allow a clear identification of the BFP. Even after reaching the BFP, slight fluctuations in the measured values may occur, which further complicate its determination. To simplify the identification and differentiate the BFP from the local extrema and plateaus, the blood flow curves were further analysed and quantified. Five blood flow curves, which were measured during previous investigations, were used for the quantisation of the blood flow curve using matlab. The result of such an analysis and quantisation of a blood flow curve is shown in Figure 14. This measurement shows the described characteristic course of the blood flow during forearm warming. The quantified curve simplifies the subsequent computer-aided identification of characteristic physiological effects such as the relative minimum and maximum as well as the BFP.

To enable a computer-aided identification of the BFP, conditions to be met were determined and tested on quantified blood flow curves and are shown in Table 3.

### 5.3. Adjustment and Determination of the Blood Flow Plateau

**Figure 15:**
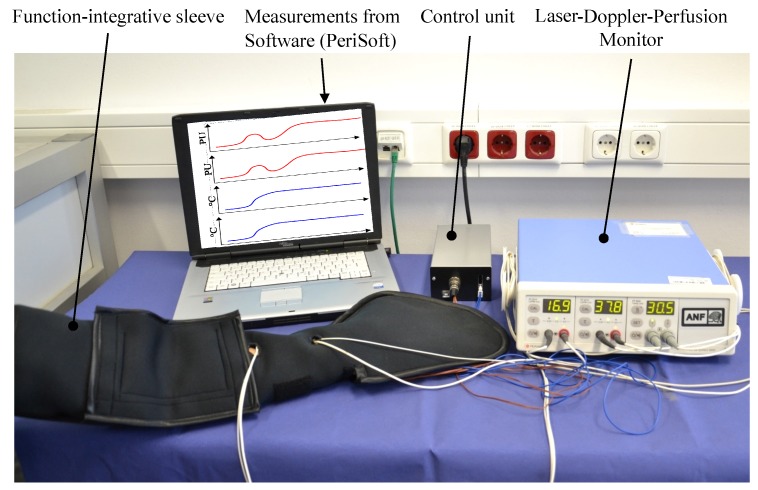
Illustration of blood flow measurement with local warming of the upper extremity using the function-integrative sleeve.

For the function test of the sleeve with regard to adjustment of the BFP, a measurement of the blood flow was carried out on a test person using the prototype. A a clinical study proving the functionality of the presented prototype is subject of future investigations comprising patients with various physical conditions. A preliminary test on a healthy 25-year-old male without vasomotor dysfunction in diabetes or other disease was conducted to give a first feedback regarding the adjustment of the targeted physiological conditions. The measurement setup is shown in Figure 15.

**Figure 16:**
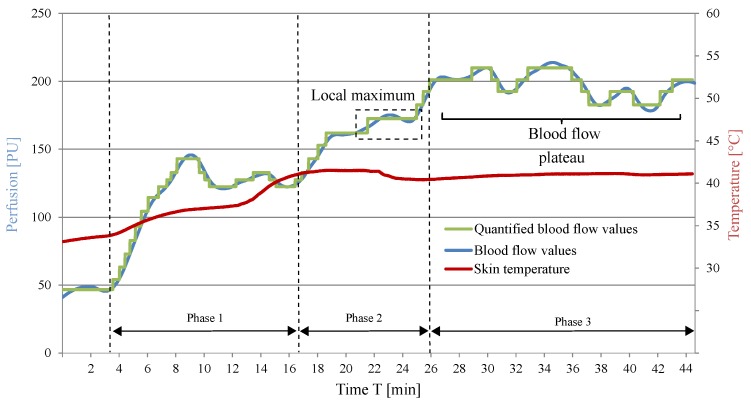
Measured blood flow curve using the function-integrative sleeve.

The blood flow values measured during the local warming of the arm are shown in Figure 16. The diagram shows a fourfold increase in blood flow over the course of the measurement due to the warming stimulus of the function-integrative sleeve. The measured maximum blood flow of 213 PU was reached 30 min after starting the local warming process.

However, after reaching its maximum, the blood flow fluctuated in the range of ±20 PU. The assessment of the influence of these fluctuations on the diagnostic procedure should be examined from a medical point of view. Subsequently, the computer-aided approach of identifying the time of maximum blood flow, described in the previous section, was applied. The short-term occurrence of a local maximum and the subsequent drop in blood flow shown in Figure 16 were not classified as the maximum blood flow from the algorithm. As a result, the point in time when the following maximum in blood flow occurred, was successfully identified.

At this point, it should be clarified that the approach was tested exclusively on the available blood flow curves. The identification of the BFP for persons whose blood flow values are subject to greater fluctuations require additional conditions than those shown in Table 3.

### 5.4. Future Works

This paper lays an emphasis on the development process of a prototype for the improvement of current diagnostic procedures regarding phaeochromocytomas and paragangliomas. The function test described in Section 5.3 was conducted to give a first feedback whether the objected physiological effects could be achieved. Among other requirements, this is necessary to plan and apply for a future clinical study. The realisation of a clinical study is prepared and it is a subject of current considerations and discussions. The objective of such a subsequent clinical study would be to prove the function of the sleeve with a higher number of healthy patients to validate the result of the functional test and relate the concentration of the tumour markers in blood before and after applying the sleeve. With regard to the homogeneity of the temperature distribution of the functional warming layer, the application of a broader HE in order to further decrease heat concentration is recommended. Additionally, the observed hot spots caused by the increased contact resistance of the contact area need to be addressed by applying conductive elastic adhesive to the textile contacting area. To increase the electrical conductivity, the conductive upper thread could be used if the tearing of the thread during the stitching process can be reduced in further investigations. The presented approach for the offline-identification of the blood flow plateau could be considered as preliminary work for future investigations comprising a clinical study as well as an analysis of blood flow characteristics during local warming. This could lead to robust and reliable online-identification of the blood flow plateau. Through the application of machine learning algorithms, a correlation could be achieved between the blood flow values measured during the forearm warming procedure and the blood flow plateau.

## 6. Conclusions

In the present paper, a prototype of a function-integrative sleeve was developed, manufactured and tested, where a functional warming layer successfully induced an increase of the local blood flow. The local warming of the forearm minimises impact of sympathetic nervous system and vasoconstriction mediated influences on measurements of catecholamine metabolites in forearm plasma, which should lead to a reduction of false-positive results for diagnostic tests reliant on such measurements. As a result, the specificity of the test procedures can be increased and costly follow-up examinations as well as unnecessary psychological stress for the patients may be minimised. Wider applications are also possible wherever forearm venous blood for measurements that can be affected by vasoconstriction-associated influences on blood flow and local release or removal of measured substances may be useful.

At first, a concept evaluation was carried out to determine whether the surface warming of the skin, using carbon fibre-based heating elements, produces the desired physiological effects on the human body. Subsequently, the design of the prototype was developed on the basis of application-specific criteria, taking, among others, anthropometric aspects into account. The integration of functional details allow the measurement of blood flow and the collection of blood samples, during the process of local warming of the patient’s upper extremity. The sleeve-shaped design enables the adaptability to different arm circumferences and thus allows it to be used by various patients with different body measurements. The preliminary investigations determined a suitable constellation of the heating elements integrated in the functional warming layer. On the basis of the geometric dimensions of the function-integrative sleeve, the arrangement of the heating elements were defined, allowing a complete warming of a patient’s arm. In the course of subsequent investigations, parameters and variables referring to the fixation process of the heating elements could be identified and adapted in favour of a more homogeneous temperature distribution on the contact surface. Following the manufacturing of the textile sleeve, an approach for the offline-identification of the blood flow plateau based on the analysis of several blood flow measurements, was developed.

Throughout a functionality test of the function-integrative sleeve, the blood flow was successfully adjusted to the level of the blood flow plateau by a fourfold increase in blood flow compared to the initial value. The conditions for an offline-identification of the beginning of the blood flow plateau were successfully tested on the blood flow curve recorded during the functionality test.

Although the optimisation of the heat distribution was successfully performed and the blood flow plateau was observed, an increase in homogeneity of the temperature distribution should be the objective of further investigations. In this context, the integration of an additional layer of high thermal conductivity as well as alternative warming mediums, such as metal-coated textiles or fluids could produce similar or improved results with respect to the homogeneous temperature distribution at the contact surface with the human body. This could lead to an improvement in comfort of the function-integrative sleeve and prevent skin irritations caused by heat-concentration at the sleeves contact layer.

## Figures and Tables

**Table 1:** The criteria for the design of the sleeve.

Criteria	Description of the Criteria
Criterion 1	Warming of the complete upper arm up to the shoulder attachment of the patient
Criterion 2	Easy adaptation of the sleeve to the patient’s arm circumference to ensure heat transfer to the patient’s skin surface
Criterion 3	Freedom of movement in the area surrounding the hand
Criterion 4	Cut-outs for two Laser Doppler Probes of the LDPM in the area of the forearm
Criterion 5	Integration of a blood sampling zone providing access to the required permanent cannula
Criterion 6	Customisability to different arm measurements

**Table 2:** Comparison of the contact resistances of textile contacting combinations with crimp contacting.

Combination	Type of Strand	γ Upper Thread	$R_{\mathrm{Cont}}$ (Textile) [Ω]	$R_{\mathrm{Cont}}$ (Crimp) [Ω]
1	round strand	conductive	0.96	1.31
2	round strand	non-conductive	1.00	1.29
3	braided strand	conductive	0.56	1.27
4	braided strand	non-conductive	0.87	1.29

**Table 3:** Listing of the conditions to be met for computer-aided identification of the BFP.

Conditions	Description
Condition 1	At least 25 min must elapse between the beginning of the warming process and the time of possible blood collection, in order to prevent premature blood sample collection.
Condition 2	The blood flow remains constant for at least 3 min after 25 min of the monitoring procedure went by.
Condition 3	If the blood flow drops after the 3 min as defined in Condition 2, the previously measured blood flow value is identified as the BFP.

## Abbreviations

The following abbreviations are used in this manuscript:

BFP	Blood flow plateau
CF	Carbon fibre
HE	Heating element
LDPM	Laser Doppler Perfusion Monitor
PU	Perfusion units
